# Intracranial hemorrhage identified after initiating therapeutic hypothermia: two case reports of neonatal hypoxic-ischemic encephalopathy due to hypovolemic shock with anemia: a case report

**DOI:** 10.1186/s13256-025-05815-w

**Published:** 2026-01-17

**Authors:** Takatoshi Murakami, Kenichi Tanaka, Ryousuke Sasaki, Shirou Matsumoto, Kimitoshi Nakamura

**Affiliations:** 1 Department of Pediatrics, Graduate School of Life Science, Kumamoto University, 1-1-1 Honjo, Chuo-Ku, Kumamoto, 860-8556 Japan; 2 Division of Neonatology, Kumamoto University Hospital, 1-1-1 Honjo, Chuo-Ku, Kumamoto, 860-8556 Japan

**Keywords:** Hypoxic-ischemic encephalopathy, Therapeutic hypothermia, Neonates, Subdural hemorrhage, Cranial ultrasound examination, Anemia

## Abstract

**Background:**

Therapeutic hypothermia for moderate-to-severe hypoxic-ischemic encephalopathy in neonates effectively improves neurological outcomes when initiated within 6 hours of birth. However, coagulopathy is a potential side effect of therapeutic hypothermia and requires careful monitoring for signs of hemorrhage. Uncontrolled hemorrhage is the primary exclusion criterion for therapeutic hypothermia. Herein, we report two cases of intracranial hemorrhage that, despite being massive enough to cause hypovolemic shock with anemia, could not be detected by cranial ultrasonography before the initiation of therapeutic hypothermia for hypoxic-ischemic encephalopathy.

**Case presentation:**

One of the two Japanese newborn cases (one male and one female, 0 years old) was delivered by vacuum extraction and the other by forceps. Both infants presented with hypoxic-ischemic encephalopathy symptoms due to hypovolemic shock with anemia, without evidence of umbilical cord rupture or ultrasonographic evidence of ongoing bleeding such as intracranial or intraabdominal hemorrhage. Therapeutic hypothermia was initiated 5 hours after birth in both cases, alongside blood transfusion. One infant (male) presented with recurrent hypotension, while the other (female) developed hydrocephalus. Subsequent computed tomography or magnetic resonance imaging revealed a subdural hematoma. In one case (the male newborn), hypothermia was discontinued due to persistent bleeding, and a craniotomy was performed for hematoma evacuation.

**Conclusion:**

These cases show that even in massive intracranial hemorrhage causing hypovolemic shock, subdural hematoma may be undetectable on bedside imaging such as cranial ultrasonography. Therefore, when therapeutic hypothermia is considered for neonates with hypoxic-ischemic encephalopathy secondary to hypovolemic shock with anemia, clinicians should be cautious and not rely solely on ultrasonography to rule out intracranial hemorrhage. Early and proactive computed tomography imaging should be performed to investigate the cause of neonatal hypoxic-ischemic encephalopathy due to hypovolemic shock with anemia.

## Background

Therapeutic hypothermia is an established treatment for infants with moderate-to-severe hypoxic-ischemic encephalopathy (HIE), with neuroprotective effects supported by both animal and systematic human research [1]. Its effectiveness depends on administration during the latent phase of cellular injury, prior to the secondary phase characterized by the onset of seizures [2]. To achieve optimal neuroprotection, therapeutic hypothermia must be initiated within 6 hours of birth [3].

Coagulopathy is a potential adverse effect of therapeutic hypothermia. Forman *et al*. reported that more than half of infants undergoing therapeutic hypothermia developed bleeding complications, including intracranial hemorrhage, pulmonary hemorrhage, gastrointestinal bleeding, and hematuria [4]. Therefore, careful monitoring of bleeding during cooling therapy is essential [5]. Uncontrolled bleeding is a major exclusion criterion for therapeutic hypothermia [6].

Neonatal hypovolemic shock with anemia commonly occurs immediately after birth [7]. Potential causes include placental or umbilical hemorrhage (for example, umbilical cord rupture and fetomaternal hemorrhage) and traumatic bleeding (for example, subgaleal and intracranial hemorrhage) [7, 8]. Although placental or umbilical hemorrhages typically cease after delivery, traumatic bleeding may persist postnatally [7]. Hypovolemic shock with anemia can lead to HIE, making affected neonates potential candidates for therapeutic hypothermia [9, 10].

Herein, we present two cases of intracranial hemorrhage that could not be detected before the initiation of therapeutic hypothermia for HIE caused by hypovolemic shock with anemia. In both cases, massive intracranial hemorrhage severe enough to cause hypovolemic shock with anemia was not identified on cranial ultrasound examination and was later confirmed by computed tomography (CT) or magnetic resonance imaging (MRI) following clinical deterioration. These cases highlight the importance of thoroughly assessing intracranial hemorrhage before initiating therapeutic hypothermia in neonates with hypovolemic shock and anemia.

## Case presentation

### Case 1

A Japanese male 0-year-old infant weighing 2586 g was born at 38 weeks gestation via forceps delivery. The patient’s mother was a healthy 32-year-old Japanese woman with no complications. His Apgar scores were 2 and 6 at 1 and 5 minutes, respectively. Umbilical cord blood pH was 7.17. At birth, the infant was pale, not crying, hypotonic, and bradycardic. He recovered with bag-and-mask ventilation, but as apnea recurred after ventilation ceased, he was intubated 35 minutes after birth. However, at 2 hours of age, the patient developed bradycardia again, requiring chest compressions. The patient was transferred to our hospital after recovery.

**Fig. 1:**
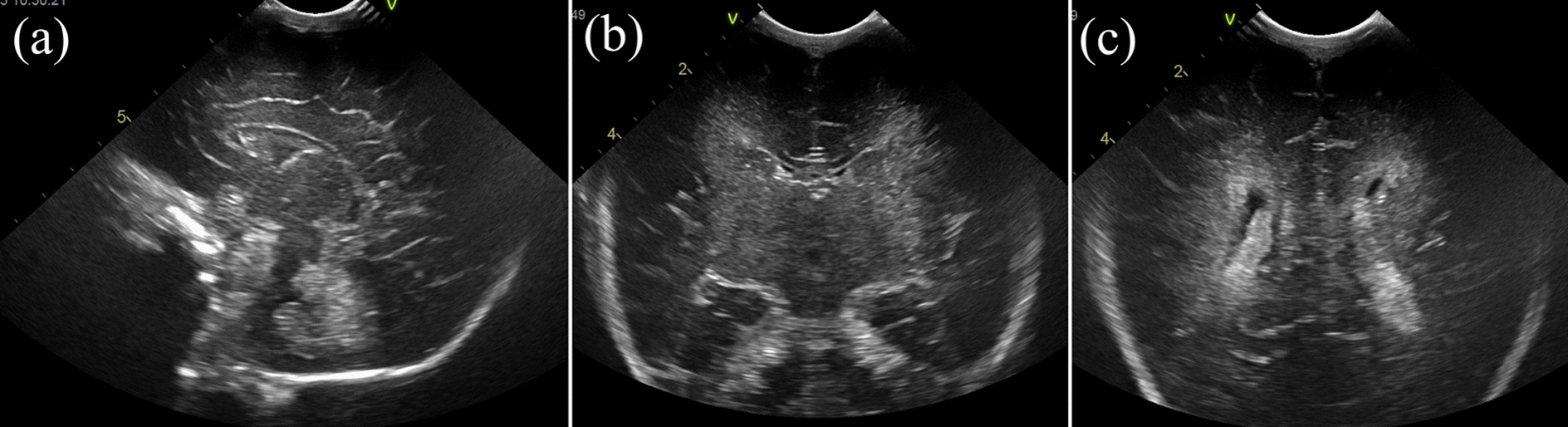
Cranial ultrasound at 3 hours after birth: **a** sagittal view, **b** coronal view (anterior horn of lateral ventricles), and **c** coronal view (posterior horn of lateral ventricles). No hyperechoic lesions indicative of hemorrhage observed

Initial laboratory findings showed hemoglobin (Hb) 4.8 g/dL, prothrombin time-international normalized ratio (PT-INR) of 3.72, serum lactate 27.5 mmol/L, and blood pressure of 35/28 mmHg. No evidence of umbilical cord rupture was found. Cranial and abdominal ultrasonography revealed no high-echo lesions suggestive of hemorrhage (Fig. 1). Thompson score was 12, indicating moderate HIE. We considered fetomaternal transfusion to be the cause of the anemia and assumed that the bleeding had already stopped. After hypotension improved following fluid resuscitation, therapeutic hypothermia with blood transfusion was initiated at 5 hours of age due to HIE.

**Fig. 2:**
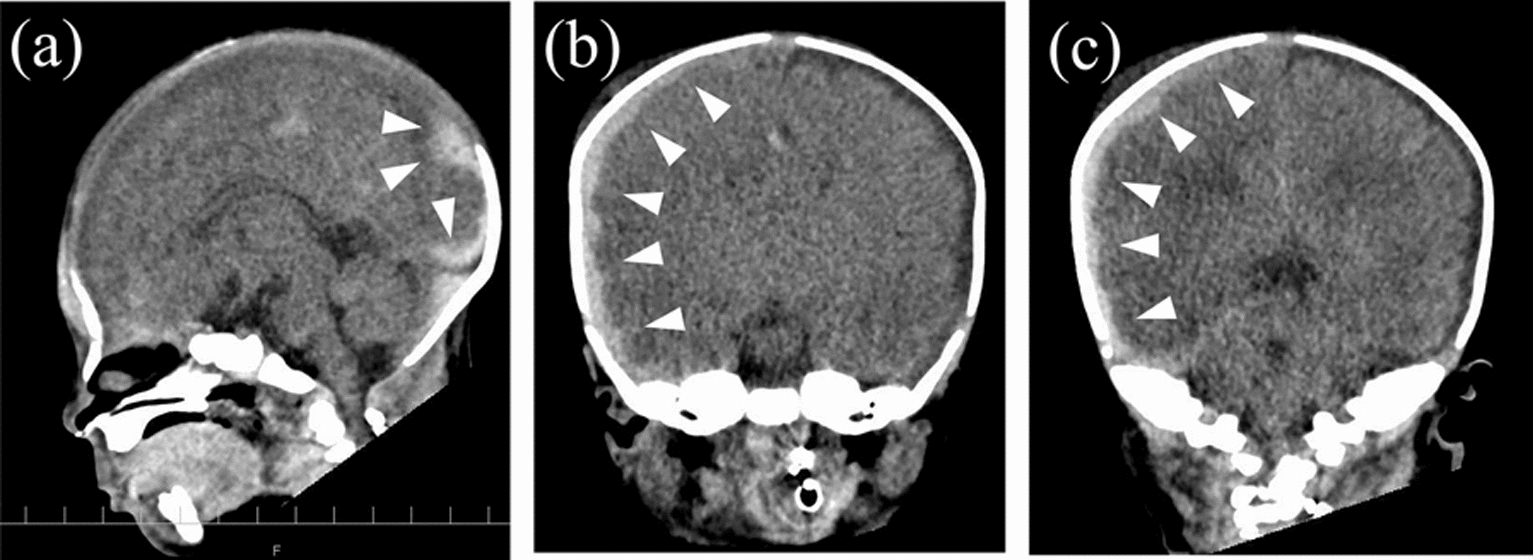
Cranial computed tomography at 14 hours after birth: **a** sagittal view, **b** coronal view (anterior horn of lateral ventricles), and **c** coronal view (posterior horn of lateral ventricles). Extensive hyperdense lesions indicative of hemorrhage present on the right side (arrowheads)

At 14 hours of age, despite undergoing a blood transfusion [red blood cell (RBC) 30 mL/kg, fresh frozen plasma (FFP) 20 mL/kg], he developed hypotension (38/28 mmHg), anemia (Hb 7.2 g/dL), and coagulopathy (PT-INR 5.4). Cranial CT revealed a subdural hematoma on the right side (Fig. 2). Therefore, therapeutic hypothermia was discontinued. Owing to brainstem compression from the hematoma and ventricular enlargement by compression of the cerebral aqueduct, hematoma evacuation was performed on day 3.

Subsequent maternal blood tests revealed α-fetoprotein (AFP) level of 258 pg/mL and hemoglobin F (HbF) level of 0.4%, ruling out fetomaternal transfusion syndrome. By day 7, 125 mL/kg of RBCs, 95 mL/kg of FFP, and 76 mL/kg of platelets were transfused. Brain MRI at 24 days of age revealed bilateral parietal and temporal lobe encephalomalacia. At 30 days of age, coagulation factors were within almost normal ranges (VIII 99%, factor IX 43%, and PT-INR 1.06). At 1 year of age, the patient remained unable to roll over or sit independently. Table 1 presents the changes in the examination results.

**Table 1:** Blood test results of case 1

	Umbilical blood	3 hours after birth	14 hours	Day 3	1 month
pH	7.18	7.12	7.33	7.47	7.34
Base excess	−9.6	−20.3	−6.5	4.6	−1.7
Lactate (mmol/L)	5.5	27.45	18.96	8.62	2.44
Hemoglobin (g/dL)	10.0	4.8	7.2	8.1	12.4
Creatine kinase (U/L)		1064	5763	3772	33
Platelets (× 10^3^/μL)		101	30	88	317
PT-INR		3.72	5.4	3.85	1.06
APTT (seconds)		153.5	72.8	38.0	38.7

*PT-INR *prothrombin time–international normalized ratio, *APTT* activated partial thromboplastin time

### Case 2

A Japanese female infant was born at 40 weeks and 1 day of gestation via vacuum-assisted delivery, with a birth weight of 2986 g. The patient’s mother was a healthy 33-year-old Japanese woman with no complications. Her Apgar score was 4 at 1 minute and 6 at 5 minutes. Umbilical cord blood pH was 7.13. At birth, she was pale, did not cry, and showed no spontaneous movement. As the condition did not improve with bag-and-mask ventilation, she was intubated 20 minutes after birth. However, at 50 minutes of age, bradycardia recurred, which required chest compressions. The patient was transferred to our hospital after recovery.

**Fig. 3:**
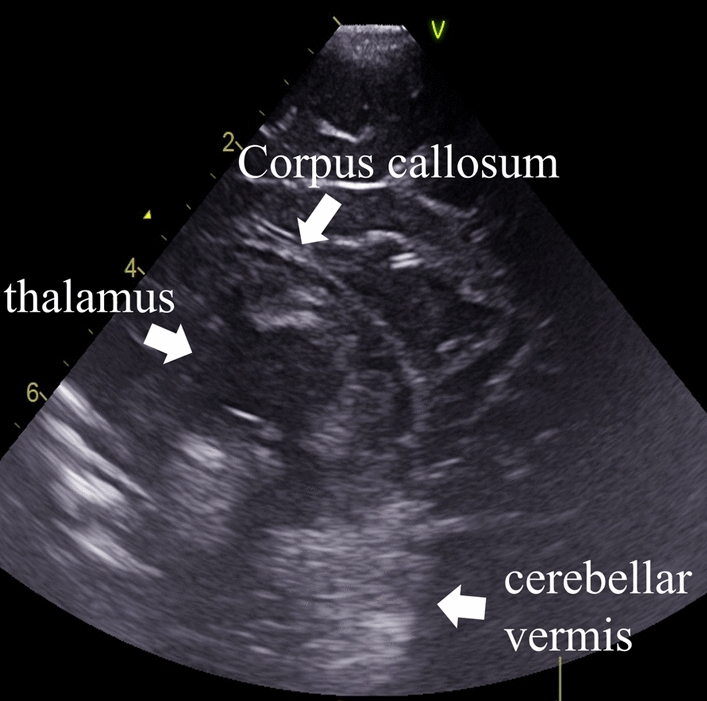
Cranial ultrasound (sagittal view) at 2.5 hours after birth. No hyperechoic lesions indicative of hemorrhage observed. The white arrows indicate normal anatomical structures (corpus callosum, thalamus, and cerebellar vermis) on the cranial ultrasound image

Her initial laboratory findings showed Hb 5.2 g/dL, PT-INR 3.18, serum lactate 18.0 mmol/L, and blood pressure 40/14 mmHg. No signs of umbilical cord rupture were observed. Cranial and abdominal ultrasonography revealed no high-echo lesions indicative of hemorrhage (Fig. 3). Thompson score was 11, indicating moderate HIE. Considering the potential for hemorrhagic shock caused by fetomaternal transfusion syndrome, we assumed that the bleeding had stopped. After hypotension improved following volume loading, therapeutic hypothermia with blood transfusion was initiated at 5 hours of age due to HIE. During the hypothermia treatment, her blood pressure remained stable, and anemia and coagulopathy improved.

**Fig. 4:**
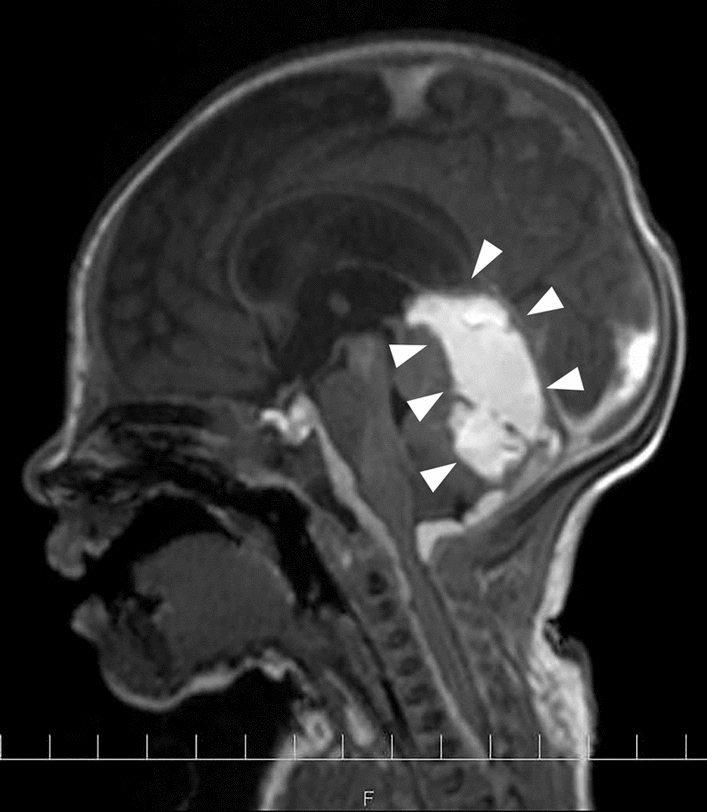
Cranial magnetic resonance imaging (sagittal view) on day 13. Infratentorial and cerebellar hemorrhages observed (arrowheads)

Bilateral ventricular enlargement was observed on day 2. No neurological symptoms, such as seizures, were observed. Therapeutic hypothermia was continued for 72 hours. Brain MRI on day 13 revealed hemorrhage in the cerebellum and posterior fossa (Fig. 4). Later, maternal blood tests revealed AFP level of 232 pg/mL and an HbF level of 0.8%, effectively excluding the possibility of fetomaternal transfusion syndrome. By day 3, 30 mL/kg of RBCs, 20 mL/kg of FFP, and 10 mL/kg of platelets were transfused.

At 1 month of age, ventricular enlargement had improved, and the hematoma was considered to have reduced in size. PT-INR was 1.05, within the normal range. Coagulation factors VIII and IX were not measured. At 1 year and 1 month of age, the infant had normal developmental outcomes. Table 2 presents the changes in the examination results.

**Table 2:** Blood test results of case 2

	Umbilical blood	50 minutes after birth	13 hours	Day 3	1 month
pH	7.13	6.92	7.39	7.416	7.445
Base excess	−9.2	−8.6	−0.8	2.3	−1.5
Lactate (mmol/L)	4.7	18.0	6.2	1.53	1.78
Hemoglobin (g/dL)	10.7	5.2	10.8	14.3	12.1
Creatine kinase (U/L)		7063	9250	2939	161
Platelets (× 10^3^/μL)		105	68	117	254
PT-INR		3.18	2.59	1.53	1.05
APTT (seconds)		56.3	43.2	45.2	37.1

*PT-INR* prothrombin time–international normalized ratio, *APTT* activated partial thromboplastin time

## Discussion and conclusion

In these case reports, despite massive bleeding causing hypovolemic shock with anemia, no intracranial hemorrhage was detected at the time of therapeutic hypothermia initiation. In one patient, therapeutic hypothermia was discontinued because of bleeding progression. This exacerbation of subdural hemorrhage, with significant neurological sequelae, may have been influenced by coagulopathy due to therapeutic hypothermia.

Subdural hematomas should be included in the differential diagnosis of neonatal asphyxia associated with hypovolemic shock and anemia. A potential side effect of hypothermia therapy is coagulopathy [5]. In case 1, hypothermic therapy may have contributed to the increased bleeding tendency and hematoma enlargement. Similarly, in case 2, the subdural hematoma was in the posterior fossa, where progressive bleeding could have led to brainstem compression and fatal outcomes if left untreated [11]. However, the diagnosis of subdural hematoma in neonates using cranial ultrasonography is difficult. In the present cases, despite massive bleeding leading to hypovolemic shock with anemia, the subdural hematoma remained undetected on cranial ultrasonography. This difficulty is likely due to acoustic interference by the bone and near-field transducer artifacts [12]. One study reported that the sensitivity of cranial ultrasound for detecting subdural hematomas was only 22.2% [13]. Blauwblomme *et al*. [14] reported 16 cases of posterior fossa subdural hematomas, none of which were detected using cranial ultrasound. These reports highlight that reliance on ultrasonography alone is insufficient for excluding intracranial hemorrhage in neonates. Clinicians should be cautious not to rule out intracranial hemorrhage solely on the basis of ultrasonography when considering therapeutic hypothermia, especially in neonates with hypovolemic shock and anemia complicated by coagulation abnormalities. In emergency situations, cranial CT is considered a more reliable imaging modality for detecting subdural hematomas [14]. In these cases, because coagulation abnormalities were present, intracranial hemorrhage should have been actively excluded by cranial CT before the initiation of therapeutic hypothermia. In fact, in case 1, intracranial hemorrhage was detected on cranial CT performed after the initiation of therapeutic hypothermia. If cranial CT had been performed at admission as part of the differential diagnosis of hypovolemic shock with anemia, the subdural hematoma might have been identified before the initiation of therapeutic hypothermia.

In case 2, an ultrasound examination was performed using only the sagittal view through the anterior fontanelle. Because the cerebellar vermis appears echogenic on ultrasound [15], a posterior fossa hematoma near the cerebellar vermis, which has high echogenicity, may not have been properly identified. If a coronal view through the mastoid fontanelle had been used, a high-density lesion due to a hematoma might have been detected [16]. Furthermore, when bilateral ventricular enlargement was observed on day 2, the possibility of hydrocephalus due to aqueductal compression by a subdural hematoma should have been considered [17].

Neonatal hemorrhages have various causes. Although umbilical cord rupture can be diagnosed relatively easily on the basis of physical findings, the diagnosis of fetomaternal hemorrhage is challenging. Although AFP measurement and Kleihauer–Betke staining of the mother are useful for detection [18], these tests often require outsourcing when the infant is transferred from a smaller hospital, leading to delays in obtaining results. Therapeutic hypothermia for neonatal HIE must be initiated within 6 hours of birth [3], and diagnosing fetomaternal hemorrhage within this timeframe remains difficult. In our cases, fetomaternal transfusion syndrome was assumed to be the cause of bleeding on the basis of the diagnosis of exclusion, as no signs of intracranial hemorrhage were observed on ultrasound and no bleeding was detected from the umbilical cord. Consequently, bleeding was considered to have stopped because the infants were separated from the placenta after birth. However, this approach may overlook other potential causes of bleeding. Given that these infants were delivered via instrumental delivery, such as forceps or vacuum extraction, subdural hemorrhage should have been considered a possible cause of anemia [12].

The absence of anemia does not rule out subdural hematoma. A study reported that 9 of 111 normal asymptomatic neonates (8.1%) had subdural hematomas [19]. Perrin *et al*. found that 10 out of 15 neonates with symptomatic subdural hematomas had Hb levels of 12.0 g/dL or higher [20]. Therefore, even in the absence of anemia, careful monitoring for the progression of subdural hematomas is necessary during therapeutic hypothermia. In fact, Wang *et al*. reported a case in which a neonate with HIE, but without anemia, developed a symptomatic subdural hematoma during therapeutic hypothermia [21].

This report has one limitation: whether it was appropriate to initiate therapeutic hypothermia—which can potentially cause coagulopathy—in infants who had already experienced severe hemorrhage. Although therapeutic hypothermia has been reported to be potentially effective in the treatment of hemorrhagic shock [22], this benefit is generally based on the assumption that bleeding has already been controlled. For example, in a rat model of hemorrhagic shock, hypothermia significantly improved survival and reduced the risk of cerebral infarction [23]. In cases of HIE associated with hemorrhagic shock due to fetomaternal transfusion syndrome or umbilical cord rupture, where bleeding ceases after birth, therapeutic hypothermia may be beneficial if both circulatory status and coagulopathy are stabilized through transfusion. In fact, the Queensland Clinical Guidelines for therapeutic hypothermia state that coagulopathy is not a contraindication if bleeding is controlled [6], and the guidelines by Takenouchi *et al*. also do not list coagulopathy as an exclusion criterion for therapeutic hypothermia [3]. However, in the present cases, the possibility of ongoing bleeding, such as subdural hematoma, should have prompted a more cautious approach to initiating therapeutic hypothermia. Shock had resolved by 5 hours of life, leaving only 1 hour before the recommended 6-hour window for initiating therapeutic hypothermia. Furthermore, because no definitive source of bleeding was identified, fetomaternal transfusion was considered the most likely cause, and head CT was not performed. This is a point of reflection in the present report. However, one must also consider the radiation risks associated with CT imaging. It has been reported that children who undergo head or neck CT scans have a higher risk of developing brain tumors compared with those without a history of CT imaging [24]. Given the limited time frame of 6 hours after birth, this risk should also be discussed with the family when considering whether to proceed with head CT. Additionally, considering that the source of bleeding was unclear, the option of withholding therapeutic hypothermia should also have been considered.

These cases highlight that cranial ultrasonography alone may not be sufficient to reliably detect or exclude intracranial hemorrhage, even in cases of massive bleeding leading to hypovolemic shock due to anemia. Therefore, when considering therapeutic hypothermia in neonates with HIE secondary to hypovolemic shock with anemia, clinicians should exercise caution and avoid excluding intracranial hemorrhage only on the basis of ultrasonography findings. For optimal therapeutic hypothermia outcomes, the early identification of the source of bleeding within the first 6 hours of birth is essential. Therefore, when implementing therapeutic hypothermia for HIE caused by hypovolemic shock with anemia, the possibility of intracranial hemorrhage should be considered, and early and proactive cranial CT should be performed before initiating therapeutic hypothermia.

## Data Availability

The datasets used and/or analyzed in the current study are available from the corresponding author upon reasonable request.

## Abbreviations

AFP: α-Fetoprotein
CT: Computed tomography
FFP: Fresh frozen plasma
HIE: Hypoxic-ischemic encephalopathy
MRI: Magnetic resonance imaging
PT-INR: Prothrombin time-international normalized ratio
RBC: Red blood cell
